# Health literacy and patient participation in multidisciplinary tumor conferences in breast cancer care: a multilevel modeling approach

**DOI:** 10.1186/s12885-019-5546-z

**Published:** 2019-04-08

**Authors:** Christian Heuser, Annika Diekmann, Christoph Kowalski, Anna Enders, Rupert Conrad, Holger Pfaff, Lena Ansmann, Nicole Ernstmann

**Affiliations:** 1 Center for Health Communication and Health Services Research (CHSR), Department of Psychosomatic Medicine and Psychotherapy, University Hospital Bonn, Sigmund-Freud-Str. 25, 53127 Bonn, Germany; 2 Center for Integrated Oncology (CIO Bonn), University Hospital Bonn, Sigmund-Freud-Str. 25, 53127 Bonn, Germany; 3 German Cancer Society e.V. (DKG), Department for Certification, Kuno-Fischer-Straße 8, 14057 Berlin, Germany; 4 Department for Research and Quality Management, The Federal Centre for Health Education (BZgA), Maarweg 149-161, 50825 Cologne, Germany; 5 Department of Psychosomatic Medicine and Psychotherapy, University Hospital Bonn, Sigmund-Freud-Str. 25, 53127 Bonn, Germany; 6 Institute of Medical Sociology, Health Services Research, and Rehabilitation Science (IMVR), Faculty of Human Sciences and Faculty of Medicine, University of Cologne, Eupener Str. 129, 50933 Cologne, Germany; 7 Organizational Health Services Research, Department for Health Services Research, Carl von Ossietzky University of Oldenburg, Ammerlaender Heerstraße 140, 26129 Oldenburg, Germany

**Keywords:** Health literacy, Multidisciplinary tumor conference, Tumor board, Patient participation, Breast Cancer, Health services research, Multilevel regression analysis

## Abstract

**Background:**

Decisions made in multidisciplinary tumor conferences (MTC) that consider patient preferences result in better patient outcomes. Furthermore, it has been shown that in some breast cancer centers in Germany, patients participate in MTCs and that participation is associated with sociodemographic and breast cancer center-related factors. Health literacy (HL) has been shown to be predictive for individual health behavior and is an important prerequisite for patient participation in healthcare. However, so far nothing is known about the association between HL and MTC patient participation. To close this gap in research, we analyzed which patient characteristics affect participation in MTCs and whether participation varies between breast cancer centers.

**Methods:**

In a prospective, multicenter cohort study, newly diagnosed breast cancer patients were surveyed directly after surgery (T1) as well as 10 weeks (T2) and 40 weeks (T3) after surgery. After descriptive analysis, t-tests were conducted, correlations for independent variables were run, and logistic multilevel regression analysis was applied to estimate the association between patient participation in MTCs at T1 and HL (HLS-EU-Q16 [1]), sociodemographic and disease-related characteristics (*n* = 863 patients) and the variation between breast cancer centers (*n* = 43 centers).

**Results:**

Descriptive results show that 6.8% of breast cancer patients took part in a MTC. The logistic multilevel regression model revealed that patients with an inadequately HL are less likely to participate in MTCs (OR = 0.31, 95%-CI = 0.1–0.9, Pseudo-R^2^ = 0.06), and participation is dependent on the breast cancer center (ICC = 0.161).

**Conclusions:**

These findings are the first to show significant differences in HL and patient participation in MTCs in a large sample of breast cancer patients. In future research on patient participation in MTCs and HL, questions concerning the organization, communication and decision-making in MTCs with and without patient participation have to be addressed, and patient and provider perspectives must be equally considered.

**Trial registration:**

Database Health Services Research, VfD_PIAT_12_001630, registered prospectively on 01.03.2012.

## Background

Oncological healthcare has been faced with many developments in recent years. Among them are *multidisciplinary care* as a process aiming to foster cooperation between professionals from a range of disciplines [2] as well as *patient centered care* as a healthcare process aiming to achieve socially and psychologically integrated care [3]. Multidisciplinary tumor conferences (MTCs) mainly represent the first development as a regular exchange between healthcare professionals [4, 5]. It remains unclear if MTCs could as well incorporate processes and structures of patient centeredness to foster decisions that explicitly consider patient preferences [6, 7]. International studies have revealed that decisions in MTCs that consider patient preferences result in better patient outcomes [8–10]. However, it has been shown that patient preferences concerning decision-making are not considered systematically in MTCs and treatment recommendations are mainly based on clinical information [11–13]. Many decisions in breast cancer care are preference-sensitive, especially in metastatic breast cancer treatment [14]. Therefore, incorporating patient preferences in MTCs is a central factor in treatment decision-making to achieve higher-quality decisions [15] and to possibly optimize adherence efficiency of MTC decisions [16].

In an effort to better take patient preferences into account, the participation of patients in MTCs has been widely discussed recently. In Germany, MTCs are part of the certification criteria of the German Cancer Society and the Medical Council of North Rhine-Westphalia [17]. The requirements of the Medical Council of North Rhine-Westphalia demand that patients should be allowed to participate in MTCs for the discussion of their own case [18]. Initial findings indicate that the active participation of patients in MTCs, e.g. concerning decision-making, is important [19, 20] because it may increase patient compliance and satisfaction [21–23]. Participating patients define themselves as collaborating actors who are able to state their preferences in the process of decision-making [24]. From the healthcare providers’ perspective, discussing therapy options with patients in the context of MTCs might be seen as a challenging part of their profession [25]. Besides potential benefits of patient participation in MTCs, studies indicate possible negative consequences for patients and providers. Patients may not fully understand the complexity of clinical information, which may increase fear regarding therapy and prognosis [26]. Moreover, the discussion between providers might be restricted. Medical experts may find it difficult to discuss a complex medical case in the presence of patients due to the need to adjust their language and to think about possible misinterpretations of their medical evaluations by the patient [27]. Further challenges might include organizational barriers, e.g. time pressure and difficult management of the patient invitation [28].

All in all, first hints concerning risks and benefits of MTC patient participation exist. To better understand how to make patient participation in MTCs beneficial, we need to know which patient subgroups are participating in MTCs in the first place, especially because we know that in some breast cancer centers in Germany, patients participate in MTCs [29]. As patients do not regularly participate in MTCs, however, little is known about the associated benefits and risks as well as participating patient subgroups. Therefore, it is important to understand which patient subgroups are likely to participate in MTCs to get a better knowledge on which patients might be more likely to find MTCs beneficial or detrimental.

In this context, one prior research shows that patients’ invitation to and participation in MTCs depends on individual sociodemographic and disease-related characteristics [30]. However, little is known about further patient characteristics. As health literacy (HL) has been shown to be predictive for individual health behavior and to be an important prerequisite for patient participation in healthcare [31, 32], it can be assumed that patients with sufficient HL are more likely to participate in MTCs. According to Sorensen et al., HL is defined as the knowledge, motivation and competence to access, understand, appraise and apply health information [33]. HL is an important factor in responding to the complex demands of modern healthcare systems, e.g., concerning the use of healthcare services, patient-provider communication, better health outcomes or disease prevention [32–36]. Previous studies found an increased prevalence of low HL especially among people with low education, older age, chronic disease or an ethnic background [1, 37]. In addition, HL does not depend on individual characteristics alone, but at the same time is a product of individuals’ capacities in an organizational setting and demands of the healthcare system, which make up organizational HL [38–40]. So far, nothing is known about the association between individual HL and patient participation in MTCs.

### Research aim

This is the first study analyzing the possible association between HL and patient participation in MTCs. The aim is to analyze the impact of individual HL, sociodemographic and disease-related characteristics as well as the impact of the variation between breast cancer centers on patient participation in MTCs.

## Methods

### Study design and sample

In a prospective, multicenter cohort study,^1^ newly diagnosed breast cancer patients were surveyed directly after (T1), 10 weeks after (T2) and 40 weeks after (T3) surgery, with three reminders sent according to Dillman’s Total Design Method [41]. Patients needed to give written informed consent to take part in the survey. The survey was approved by the Ethics Committee of the Medical Faculty of the University of Cologne. Data were collected from 2013 to 2014 using standardized self-report measures in written questionnaires. Breast cancer centers were recruited by randomly sampling 98 out of 247 German breast cancer centers meeting the criteria of the German Cancer Society and the German Society for Senology. In total *n* = 56 breast cancer centers took part in the study and *n* = 43 were included in the analysis of the study; this discrepancy is due to missing or insufficient patient data in some centers. Patient inclusion criteria were inpatient surgery for newly diagnosed breast cancer (C50.xx, D05.xx) performed between February 01 and August 31, 2013, at least one malignancy and at least one postoperative histological evaluation. In total, 1395 patients took part in the study (response rate = 87.7%), with *n* = 863 (61.9%) patients included in the analysis, with the discrepancy due to missing data in dependent and independent variables.

### Instruments and variables

All measures and instruments used in the questionnaires were pre-tested in a pilot study as described elsewhere [42, 43]. All items used in this manuscript were measured in T1. Patients who filled out the item “Have you been offered the opportunity to participate in a tumor conference?”, (1 = Yes and I took it; 2 = Yes and I did not take it; 3 = No) were considered. The item was used as dependent variable in order to measure the participation in MTC (0 = “No” and “Yes and I did not take it”; 1 = “Yes and I took it”). HL was measured with the validated HLS-EU-Q16 questionnaire. HLS-EU-Q16 was categorized into ‘sufficient’, ‘problematic’ and ‘inadequate’ categories according to common standards [1] (Cronbach’s Alpha 0.90). Data on sociodemographic patient characteristics were assessed in the patient survey with the help of self-reported items (formal education, age, health literacy, living with partner, native language, health insurance status). Data on clinical patient characteristics were provided by the clinical personnel (UICC stage). Table 1 shows the descriptive results of the 863 patients and 43 breast cancer centers included in the model.

**Table 1:** Descriptive results of the *n* = 863 patients

Variables	Response trait	*n* (%)
Dependent variable: participation in MTC	No	804 (93.2)
	Yes	59 (6.8)
Highest education level achieved	No school education	62 (7.2)
	Lower secondary school education	503 (58.3)
	Intermediate secondary school education	101 (11.7)
	Entrance certificate for a university of applied sciences / University entrance certificate	197 (22.8)
Age	18–39	36 (4.2)
	40–49	193 (22.4)
	50–59	273 (31.6)
	60–69	204 (23.6)
	≥70	157 (18.2)
Health literacy	Inadequate	139 (16.1)
	Problematic	287 (33.3)
	Sufficient	437 (50.6)
Living with partner	No	222 (25.7)
	Yes	641 (73.2)
Native language	German	827 (95.8)
	Other	36 (4.2)
Health insurance status	Statutory	659 (76.4)
	Private	89 (10.3)
	Statutory with additional private insurance	115 (13.3)
UICC stage	Stage 0 / I	441 (51.1)
	Stage II	300 (34.8)
	Stage III / IV	122 (14.1)
*n* patients		863
*n* breast cancer centers		43

### Analysis

Firstly, the data were analyzed descriptively. Secondly, intercorrelations among the independent variables were checked for multicollinearity. Lastly, the associations between patient participation in MTCs and HL, sociodemographic and disease-related characteristics were analyzed using two-level random intercept hierarchical logistic models. As the data were hierarchically structured, i.e., individual patient data were nested in breast cancer center clusters, multilevel modeling was used to account for clustering [44, 45]. In a first step, a two-level model without predictors (null model) was fitted in order to calculate the intraclass correlation coefficient (ICC null model). The ICC indicates the proportion of variance in the dependent variable that is attributable to differences between breast cancer centers. In a second step, patient characteristics were added as predictors at the patient level. The resulting odds ratios (OR) and 95% confidence intervals (95%-CI) were standardized via standard deviations to allow comparisons of effect sizes. No imputations were performed for missing data. Due to listwise deletion for the dependent variable and all independent variables, individual data of *n* = 863 patients and organizational data of *n* = 43 centers were included in the model. All analyses were conducted with STATA version 15.

## Results

### Descriptive analyses

According to the survey, 59 patients (6.8%) took part in MTCs. For detailed descriptive results of the sample see Table 1.

Sufficient HL scores were found in 61% of the participating patients, whereas 32.3% exhibited problematic and 6.8% inadequate HL. Of the non-participating patients, 49.9% had sufficient HL, 33.3% problematic HL and 16.8% inadequate HL. T-tests revealed that the HL status of participating patients (mean 2.54) and non-participating patients (mean 2.33) significantly differed (t = − 2.12, *p* = .05). T-test results are shown in Table 2. The proportion of patients participating in MTCs in the 43 breast cancer centers ranged from 0 to 75%.

**Table 2:** T-test results with HL status and MTC patient participation

HL	Participating patients	Non-participating patients
Inadequate	6.8%	16.8%
Problematic	32.3%	33.3%
Sufficient	61.0%	49.9%
	Mean: 2.54 SD: 0.62 95%-CI: 2.38–2.70	Mean: 2.33 SD: 0.75 95%-CI: 2.28–2.38

### Multivariate analyses

No multicollinearity was found between the independent variables. The logistic multilevel regression model revealed that HL is significantly associated with the participation of breast cancer patients in MTCs (Pseudo-R^2^ = 0.06). Patients with inadequately HL are less likely to participate in MTCs (OR = 0.31, 95%-CI = 0.1–0.9) than patients with sufficient HL. Concerning other sociodemographic or disease-related characteristics, no significant associations were found in the model. The ICC shows a value of 0.161 (16.1%) and the ICC null model of 0.149 (14.9%) indicating that 14.9% of the variance in the dependent variable (patient participation on MTC) is associated to differences only between breast cancer centers (level 2). All results are shown in Table 3.

**Table 3:** Logistic multilevel hierarchical regression model with participation in the MTC as the dependent variable

Variables	Response trait	OR^1^	95%-CI^2^
Highest education level achieved	No school education	1.93	0.74–5.31
	Lower secondary school education (Ref.)	1.00	
	Intermediate secondary school education	1.74	0.75–3.97
	Entrance certificate for a university of applied sciences / University entrance certificate	1.12	0.54–2.34
Age	18–39	0.39	0.10–3.15
	40–49	0.69	0.30–1.56
	50–59 (Ref.)	1.00	
	60–69	0.80	0.38–1.69
	≥70	0.93	0.42–2.08
Health literacy	Inadequate	**0.31**	**0.11–0.93**
	Problematic	0.85	0.47–1.56
	Sufficient (Ref.)	1.00	
Living with partner	No	1.00	
	Yes	0.92	0.48–1.75
Native language	German	1.00	
	Other	1.89	0.49–7.23
Health insurance status	Statutory (Ref.)	1.00	
	Private	0.74	0.26–2.12
	Statutory with additional private insurance	0.95	0.39–2.27
UICC stage	Stage 0 / I (Ref.)	1.00	
	Stage II	0.83	0.45–1.53
	Stage III / IV	0.39	0.13–1.18
*n* patients		863	
*n* breast cancer centers		43	
ICC^3^ (ICC null model)		**0.161 (0.149)**	

^1^Standardized odds ratios (OR)

^2^95%-confidence intervals (95%-CI)

^3^Intraclass correlation coefficient (ICC)

Significant results in bold (*p* < 0.05)

## Discussion

The aim of the study was to examine how HL, sociodemographic patient characteristics and disease-related patient characteristics are associated with patient participation in MTCs and to what extent patient participation in MTCs varies between breast cancer centers. The logistic multilevel regression model showed that significant differences in participation exist between HL level groups and between breast cancer centers. To the best of our knowledge, our findings show this association for the first time. In the following sections, specific aspects of these results are discussed in detail.

### Patient characteristics

Descriptive results show that 6.8% of breast cancer patients in German breast cancer centers took part in MTCs, which is a slightly lower percentage than in other studies based on data from North Rhine-Westphalia [30]. This may be due to the two different requirement catalogues of breast cancer centers in Germany: The Medical Council of North Rhine-Westphalia demands that patients should be allowed to participate in MTCs for the discussion of their own case, which led to a call for invitation to MTCs. In contrast, the German Cancer Society (Germany-wide) does not demand patient participation in MTCs.

The descriptive and multivariate results of our analyses reveal that patients with an inadequately HL are less likely to participate in MTCs. Our results are in line with research on the implications of individual HL on health-related behavior [32, 34]. Higher HL enables patients to better communicate with healthcare professionals and to be more involved in diagnosis and treatment [36, 46]. This may ultimately lead to better health process measures and health outcomes [1, 33, 35].

As patients’ formal education, age, native language, partner status and health insurance showed no significant association in the model, it can be concluded that in the here presented sample of breast cancer survivors sociodemographic characteristics are not associated with patient participation in MTCs. This might be seen as contradictory to common findings as highly educated and/or younger patients would be more likely to participate in MTC due to greater HL, greater coping skills and stronger preferred involvement in decision-making [47, 48]. Prior findings from Ansmann et al. on patient participation in MTCs show opposite findings as well [30]. One explanation might be the lack of a definition of “MTC” given in the questionnaire potentially leading to a misunderstanding of the questionnaire item “participation in MTC”. This might indicate that patients with lower education possibly have a greater tendency to respond socially desirable (MTC participation “yes”). Another reason might be an inconsistent invitation of patients from healthcare providers as patients might be selected based on sociodemographic and/or disease-related characteristics. Lastly, missing data has to be taken into account: patients with lower education, higher age, inadequately health literacy, native language not “German” and statutory health insurance status descriptively show a greater amount of missing data.

The model shows no significant associations for the UICC stage. This result stands in contrast to prior findings [30] and to the hypothesis that patients with a UICC stage III/IV take part more frequently in MTCs because in advanced disease stages, more treatment options can be discussed and therefore patients may think that they can benefit more from stating their preferences in the decision-making process. The absence of differences in our study might be explained, on the one hand, by the small number of patients in the categories UICC stage III/IV. On the other hand, from the perspective of severely compromised patients with UICC stage III/IV, the physical and emotional effort associated with participating in MTCs may outweigh potential benefits of their involvement in the decision-making process.

### Breast cancer center variation

Prior findings showed that invitations extended to patients to take part in a MTC and actual participation rates vary significantly between breast cancer centers [30]. The ICC for the null model implies that 14.9% of the variance in participation in MTCs is attributable to the breast cancer center. The rather high ICC underlines the fact that providers’ attitudes towards patient participation in MTCs as well as the associated processes before, during and after MTCs differ quite strongly between breast cancer centers. This leads to research implications regarding the examination of specific risks and benefits emerging from patient participation in MTCs for patients and providers across various centers. After this examination the question can be raised whether MTC participation and the processes to ensure participation might be one aspect of organizational HL [49] in the sense of patient centeredness.

### Limitations and strengths

When interpreting these findings, some limitations, strengths as well as future research and practice implications have to be considered. As a *limitation*, the observational design with mainly self-reported items has to be taken into account; it might contain a possible systematic error in the variance of the dependent variable. Furthermore, three patient selection processes might have taken place: firstly, only women were included in the analysis, secondly, although we are not able to test it, we suggest that healthier patients might have filled out the questionnaire more often and lastly, the characteristics of patients with missing data (see “Patient characteristics”). Another source of overestimation of associations might be the common method bias. Overall, no causal effects can be formulated due to the observational design. A *strength* of the study is the nationwide random sample of breast cancer centers and patients. Furthermore, we considered the nested data structure with the multilevel modelling approach. Additionally, the multilevel regression model can be seen as an advanced statistical method combining many patient variables in one model.

### Implications

As *research implications* for this study, four main aspects have to be taken into account. (1) In general, our results supply no evidence regarding the risks and benefits of patient participation in MTCs for patients and providers. Concerning future study designs, a triangulation [50] of different qualitative and quantitative methods is needed to better equally address the perspectives of patients and providers on MTC participation. Such a mixed-methods study [51] on risks and benefits might include patient experiences of MTC participation and their psychosocial situation as well as provider perspectives of possible opportunities associated with and barriers to patient participation in MTCs. In addition, a future study might be able to clarify the association between the sociodemographic characteristics included here and the likelihood of participation. Ideally, future research would benefit from an interventional study design. (2) Research on patient participation in MTCs and HL differences has to give greater consideration to healthcare processes. Patient-provider communication and decision-making in MTCs have to be additionally addressed with the help of different concepts [46]. This might include, e.g. the patients’ perspective on organizational patient-centred efforts in MTCs in order to analyze healthcare organizations’ responsiveness to patients’ individual needs in decision-making and patient-provider communication during MTC. This seems to be an important aspect of HL for future research as communicational processes and skills are considered increasingly important in healthcare. A research-guiding hypothesis in this context is the above-mentioned question of whether MTCs in their common form represent processes and structures of patient centeredness, because they mainly consist of decisions which consider patient preferences [6, 52]. This might include the question of whether the participation of patients in MTCs is an effective tool to realize patient centeredness. Therefore, relevant aspects (see (1)) have to be compared in MTCs with and without patient participation. (3) The interesting interaction between HL and sociodemographic characteristics, which is increasingly discussed in Germany [37, 53], should be considered more strongly in MTC research. (4) As the missing data contains patient subgroups which represent potential vulnerable groups in healthcare (low education, higher age, low health literacy) it is important for future studies to follow strategies reaching these patients. This might include the adjustment of survey instruments in simple language, the use of qualitative research approaches instead of using standardized quantitative instruments which are potentially difficult to fill out, and the cooperation with organized patient groups (e.g. self-help groups) in order to integrate research questions focusing on specific (information) needs or (emotional) concerns, e.g. the procedures of a MTC.

As *practical implications* for level 2 (organization and providers), it is worth considering factors that may possibly encourage or hinder the adoption of patient participation in MTCs. This may include the following aspects concerning the organization of MTCs: the selection and the consistent or inconsistent invitation of patients, a verbally and written definition of and invitation to the MTC which is easily understandable for all patients, the duration of the MTC per patient, the number of discussed cases in one MTC, interruptions during MTCs, or documents and technical aids. Furthermore, processes in the MTCs like the interaction between the providers as well as the interaction between providers and patients must be studied. Lastly, to include provider and breast cancer center manager attitudes towards patient-centered approaches, the provider experiences concerning the involvement of patients in the decision-making process is important. For level 1 (patients), the subjective experiences of patients in MTCs are important in order to explore the potential risks and benefits of participation. This may include patient expectations, concerns, and fears before and after the MTC and patient experiences concerning the decision-making.

## Conclusions

This study has highlighted a significant research gap concerning the individual and organizational determinants of patient participation in MTCs among breast cancer patients. The findings show that significant differences in MTC patient participation exist between patient groups (individual HL) and between breast cancer centers, and hence demonstrate the necessity of more research in this field. Overall, future research and practice should answer the questions regarding risks and benefits of patient participation in MTCs. Deeper insight into the feasibility of patient participation in MTCs, possible subgroups of patients that might benefit from participation as well as the quality of the decision-making process would be beneficial in the development of specific recommendations for patients and providers in MTCs.

## Footnotes

1 PIAT: Strengthening patient competence: Breast cancer patients’ information and training needs (Funding: The German Federal Ministry of Health; Carried out by: Institute for Medical Sociology, Health Services Research, and Rehabilitation Science (IMVR))
